# Molecular insights into the anti-inflammatory efficacy and HPLC analysis of hedera helix leaf extract

**DOI:** 10.4314/ahs.v25i2.39

**Published:** 2025-06

**Authors:** Khaled Qabaha, Jehad Abbadi, Fuad Al-Rimawi

**Affiliations:** 1 Department of Medical Laboratory Sciences, Faculty of Allied Health Sciences, Arab American University in Palestine, Jenin, Palestine; 2 Biology Department, Faculty of Science and Technology, Al-Quds University, P.O. Box 20002, Jerusalem, Palestine; 3 Chemistry Department, Faculty of Science and Technology, Al-Quds University, P.O. Box 20002, Jerusalem, Palestine

**Keywords:** Anti-inflammatory, Herbal drugs, Phenolics

## Abstract

**Aim:**

The Araliaceae family member Hedera helix L is well-known in traditional medicine for its ability to effectively treat a wide range of illnesses. This study uses High-Pressure Liquid Chromatography (HPLC) with a Photodiode array detector (PDA) detector to examine the phytochemical composition of the ethanolic extract obtained from Hedera helix leaves and explores its potential as an anti-inflammatory agent. Specifically, flavonoids and polyphenolic compounds are the focus of this analysis.

**Methods:**

Ethanol was used to extract compounds from ivy leaves. Real-time reverse-transcriptase polymerase chain reaction (RT-PCR) was used to investigate its anti-inflammatory properties using lipopolysaccharide-pretreated white blood cells. Phytochemical analysis of the ivy leaf extract was conducted using the reversed-phase HPLC with PDA.

**Results:**

High-Pressure Liquid Chromatography of the ethanolic extract revealed the presence of numerous polyphenolic compounds, three of which were successfully identified as 4-Hydroxyphenyl acetic acid, rutin, and hesperidin. Furthermore, the extract demonstrated significant anti-inflammatory properties, notably inhibiting the gene expression of Interleukin-6.

**Conclusions:**

The results of this investigation underscore the anti-inflammatory potential of Hedera helix extract. Various polyphenolic compounds and flavonoids were detected in the ethanolic extract, three of which were successfully identified. These compounds are presumed to be responsible for the observed activities. Further studies are necessary to elucidate the specific mechanisms of action.

## Introduction

Real-time reverse-transcriptase polymerase chain reaction (RT-PCR) has gained widespread popularity in quantitative molecular biology^1^. It is now recognized as a standard technique for assessing gene expression, which can influence the pathophysiology of diseases or predict health outcomes. In RT-PCR experiments, total RNA is extracted from tissues or cells under various conditions, such as exposure to different radiation levels, dietary intake, or treatments, as well as diverse physiological and pathological states like age, pregnancy, lactation, and tumorigenesis. This RNA is then reverse-transcribed and amplified using DNA polymerase. Specific primers amplify target genes during the RT-PCR analysis process, which typically involves denaturation, annealing, and extension phases in each cycle. Through successive cycles, one copy of the target gene undergoes doubling, then quadrupling, and eventually exponential amplification, resulting in a significant increase in copies^2^,^3^.

The use of herbal medicine and products made from plants for treating various pathological illnesses has increased significantly in recent decades. They have numerous health benefits and lack harmful or severe side effects compared to synthetic chemicals^4^.

Dry Ivy leaf extract, scientifically known as Hedera helix L. in the Araliaceae family, is extensively employed to alleviate symptoms associated with acute or chronic inflammation of the respiratory system, particularly when coupled with coughing^5^,^6^. Studies have demonstrated its anti-inflammatory properties in mice with chronic-induced asthma, corroborating its efficacy in managing respiratory conditions^7^.

The bioactive compounds found in Hedera helix leaves have drawn more attention lately. Early research examined H. helix saponins' antibacterial^8^ and antifungal properties^9^. The primary component of the cell wall of gram-negative bacteria is called lipopolysaccharides (LPS). Only after bacterial lysis or any other external damage is it released into the extracellular medium to carry out its specialized bioactivity. Due to their specific harmful effects, LPS are referred to as endotoxins. Lipopolysaccharides are frequently employed as an effective external agent that causes inflammatory responses either in vivo or invitro^10^.

Pro-inflammatory cytokines, such as Intelukin-6 (IL-6), are produced and elevated after LPS treatment of polymorphonuclear cells via NFK-B cascade signal pathways^11^. Interlukin-6 is secreted by many cells, including human polymorphonuclear leukocytes as neutrophils, lymphocytes, and monocytes^12^. Its main function is to mediate and regulate immune response against bacteria and other germs^13^.

In the context of inflammation, interleukin-6 plays a major pro-inflammatory effect as it contributes to the initial stages of the inflammatory response^14^. When there is tissue damage or infection, immune cells release IL-6 to trigger a cascade of immune responses. It helps recruit other immune cells to the site of inflammation, increases the production of acute-phase proteins by the liver (such as C-reactive protein), and stimulates the production of other pro-inflammatory cytokines like IL-1 and TNF-alpha. These responses help the body mount a defense against potential threats^5^,^15^.

To the best of my knowledge, this work has not been conducted before in the literature. Therefore, the current research aims to investigate the anti-inflammatory effect of the ethanolic extract of the Ivy plant by Real-time reverse-transcript PCR and to analyze the extract for its polyphenolic compounds and flavonoids by HPLC-PDA.

## Materials and methods

### Plant Collection

Hedera helix plants were collected in June 2022 from the wild in Ramallah/West Bank. The leaves were dried in the shade for one week, and then finely ground using a mortar and pestle. The plant was classified by Khalid Sawalha and preserved in the Herbarium of Al Quds University under voucher number AQU-H-2014-0154

### Extraction

Five grams of dried Hedera helix leaves were soaked in 50 ml of ethanol (99%) and subjected to ultra-sonication for two hours. After suction filtration, the solvent was evaporated using a rotary evaporator operating at low pressure after filtering the mixture. The resulting viscous crude extract is transferred into small glass screw-capped bottles, the bottles were placed in a refrigerator for further examination^16^. For HPLC analysis, the extract was dissolved in 99% ethanol to a concentration of 1.0 mg/ml. The analysis (HPLC and anti-inflammatory activity) was performed in triplicate for each of the three samples.

### Determination of Phytochemicals by HPLC-PDA

High-pressure liquid chromatography equipped with a photodiode array detector (HPLC with PDA) was used for the analysis of 27 compounds of phytochemicals (phenolic compounds and flavonoids). Hypersil RP BDS C18 column (Thermo Scientific, 150 x 4.6 mm, 3 µm) was used, with a flow rate of 0.7 mL/minute using the gradient elution method. Table 1 shows the mobile phase composition and the gradient elution program used to detect main components, where (A) is 1.0% acetic acid and (B) is acetonitrile. The PDA range was set from 210 to 400 nm, while the column temperature was set to 25°C. The injection volume was set to 20 µL. All samples were filtered through a 0.45 µm disposable filter.

**Table 1:** Mobile phase composition

Time	B (1.0 % HAc)	C (ACN)
0	93	7
40	80	20
50	65	35
70	40	60
75	10	90
78	93	7
80	93	7

The following standards: gallic acid, 3,4-dihydroxybenzoic acid, 3,4-dihydroxyphenylacetic acid, chlorogenic acid, 4-hydroxyphenyl acetic acid, vanillic acid, caffeic acid, syringic acid, p-coumaric acid, ferulic acid, sinapic acid, rutin, verbascoside, quercetin, trans-cinnamic acid, and kaempferol were prepared using a solvent of 20% ethanol with a concentration of 0.25 mg/mL. A standard mixture was made by mixing 1.0 mL of each standard solution into a 25 mL volumetric flask that was made up of volume with the same solvent.

### Cell culture

Five milliliters of freshly transfused whole blood were utilized for the isolation of polymorphonuclear cells. Under sterile conditions, the blood was mixed in a 1:1 ratio with phosphate-buffered saline (PBS) in equal volumes. Subsequently, three milliliters of Ficoll-Histopaque were carefully pipetted into a 15-milliliter conical tube. The PBS and blood mixture were gently poured into the tube, which was then centrifuged at 400 xg for 20 minutes. Following centrifugation, the polymorphonuclear cells (PMNCs) were aspirated, and three wash cycles were performed using 10 ml of PBS in a 12-milliliter conical tube, centrifuging at 100 xg for ten minutes each time. Finally, the cells were separated and utilized in our investigation. The separated PMNCs were enriched by adding heat-inactivated 10% Fetal Bovine Serum (FBS) and 100- µg ml-1 streptomycin, 100 Uml-1 penicillin to Roswell Park Memorial Institute (RBMI) medium. The mixtures were incubated in a 12-well tray for 24 hours at 37°C with 5% CO2. Each well was used to incubate 1 ml of the mixture, which includes 1 million of the cells. Different extract concentrations were applied to cells that had been stimulated by lipopolysaccharide (1 µg/well).

### Trypan blue exclusion cytotoxicity test for cell viability

The cytotoxicity of the Ivy leaves extract was assessed using the trypan blue exclusion test. Trypan blue was diluted to become 0.4% in normal saline and then mixed with an equal amount of the cell suspension. Three hundred µg of the extract was added to the mixture. We used a Hemocytometer to count the cells. Dead cells were blue and the live cells were unstained.

### RNA Extraction

RNA was extracted from cultured human PMNCs. After the media was removed, cells were pelleted after centrifugation at 55,000 xg for 15 minutes. Pellets were lysed using 1ml Trizol (TRI) reagent per 10 cm2 plate, collected in Eppendorf tubes, and allowed to stand for 5 minutes at room temperature.

For each tube, 0.2 ml chloroform was added, vortexed for 15 seconds, and left to stand for 5–15 minutes at room temperature. The mixture was centrifuged at 12,000-xg for 15 minutes at 4 °C. The mixture was separated into three layers, and the aqueous supernatant colorless layer containing RNA was transferred to a new Eppendorf tube. For each tube, 0.5 ml ice-cold 2-propanol was added and allowed to stand for 5-10 minutes at room temperature before centrifugation at 12,000 xg at 4°C. The RNA was detected as a white precipitate in the bottom of the tube. The RNA pellet was washed with 1 ml 70% ethanol and centrifuged at 7,500-xg for 5 minutes at 4°C. The supernatant was removed and the RNA pellet was air dried for 5 minutes.

### cDNA synthesis

cDNA was synthesized using a maxima cDNA synthesis kit according to the manufacturer's recommendation using 1µg RNA/sample. The reaction was carried out in an **APPLIED BIOSYSTEMS** thermocycler according to the following protocol; incubation at 25°C for 10 minutes followed by incubation at 50°C for 15 minutes and the reaction was terminated at 85°C for 5 minutes.

### Gene expression evaluation using RT-PCR

The level of Interlukin-6 (IL6) gene expression using the actin gene as reference was quantitated by Real-time PCR using 2X SYBR™ Green PCR Master Mix and the synthesized cDNA after dilution 1:1with nuclease-free water and the relevant primers after dilution to 10 µM. Two sets of primer pairs were used in RT-PCR to quantify the expression levels of IL6. The primers are illustrated in Table 2.

**Table 2:** Primers list in Real-time PCR

IL-6	Forward	5′- CCTCCAGAACAGATTTGA GAGTAGT-3′
	Reverse	5′-GGGTCAGGGGTGGTTATTGC-3′
B-actin	Forward	5′-TGAGACCTTCAACACCCCAGC-3′
	Reverse	5′- ACAGCTTCTCCTT AATGTCACGC- -3′.

The samples were arranged in a 96-well real-time PCR plate, sealed, and centrifuged. The reaction was performed using APPLIED BIOSYSTEMS 7500 RT PCR machine according to the following program; Initial denaturation at 95°C for 15 seconds followed by 40 cycles of 95°C for 30 seconds, annealing at 600C for 60 seconds, elongation at 600C for 60 seconds and dissociation curve at 950C for 15 sec.

### Statistical analysis

Three samples of Ivy plant (Hedera Helix) were independently analyzed and all of the determinations were carried out in triplicate. The results are expressed as means ± standard deviations. All statistical analyses were carried out using SAS (SAS Institute Inc., Cary, USA, Release 8.02, 2001). Comparisons of means were carried out using the GLM procedure, treating the main factor separately using one-way analysis of variance (ANOVA). The Bonferroni procedure was employed with multiple t-tests to maintain an experiment-wise of 5%.

## Results

### Cytotoxicity of the Ivy Leaves extract

Compared to the control group, Ivy leaf extract has no discernible impact on the viability of the PMNCs. These outcomes are shown in Table 3. These findings suggest that any potential decrease in cytokine production is not caused by the demise of PMNCs.

**Table 3:** Effects of LPS and Ivy leaf extracts on the viability of PMNCs

Contents	Viability%
Cells only	96% ± 1.5
Cells with LPS	94%± 1.7
Cells with LPS and 300 µg/ml extract	93.2±

Results are expressed as average – standard deviation (n = 3).

LPS, lipopolysaccharide; PMNCs, polymorphonuclear cells.

### Anti-inflammatory activity of The Ivy leaf extract

Using Real-Time PCR, the anti-inflammatory potential of the Ivy leaf extract was examined concerning the expression of the IL-6 gene. As a control, the b-actin gene was employed. Table 4 presents the results.

**Table 4:** Interleukin-6 gene expression upon the effect of the Ivy Leaves extract

PMNCs only	Fold change of IL-6 Gene expression
Control ( B-actin)	1
PMNCs with LPS	614 ± 1.7
PMNCs with 150 µ g extract and LPS	181 ± 1.5
PMNCs with 300 µ g and LPS	33 ± 1.2

Results are expressed as average – standard deviation (n = 3).

Ivy leaf extract contains many bioactive compounds, such as saponins, flavonoids, and other polyphenols, which are expected to contribute to its potential anti-inflammatory effects^17^.

### HPLC chromatogram of standards

**Figure 1:**
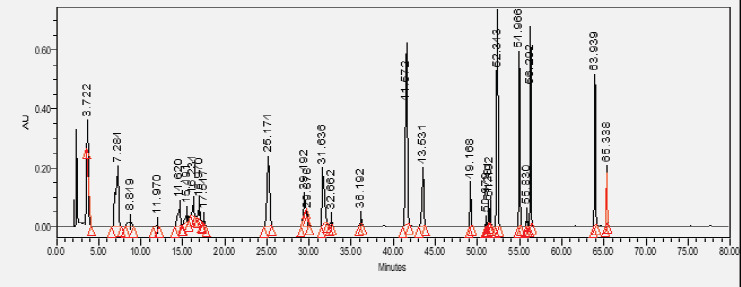
chromatogram of the 27 standards of phenolic compounds and flavonoids using the developed method in this study at 330 nm

Figure 1 shows a chromatogram of the 27 standards of phenolic compounds and flavonoids using the developed method in this study at 330 nm. Table 5 shows the retention time and UV spectrum of these polyphenolic standards separated using the method.

**Table 5:** Retention time and UV spectrum of the polyphenolic standards separated using the method

	Name	R.T. (min)	UV scan
1	Gallic acid	3.3	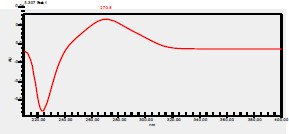
2	3,4-Dihydroxybenzoic acid	6.67	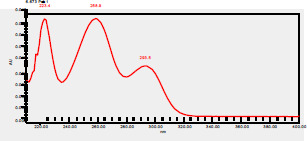
3	3,4-Dihydroxyphenyl acetic acid	8.19	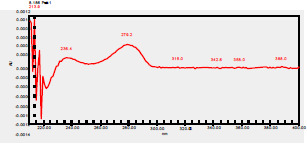
4	Chlorogenic acid	11.07	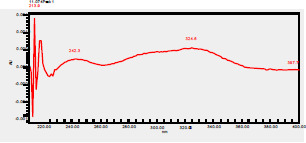
5	4-Hydroxyphenyl acetic acid	13.64	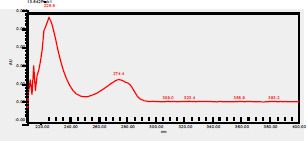
6	Vanillic acid	14.61	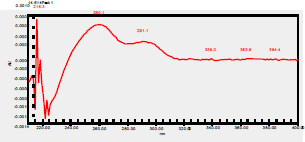
**7**	Caffeic acid	15.05	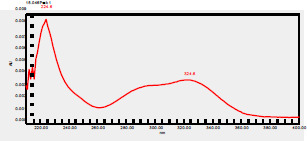
8	Syringic acid	16.11	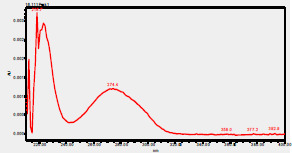
9	Isovanillic acid	16.58	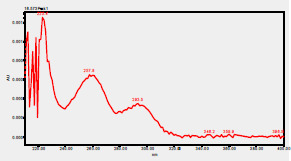
10	p-Coumaric acid	23.53	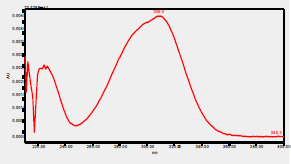
11	Ferulic acid	28.05	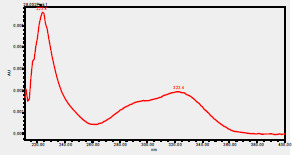
12	Sinapic acid	28.54	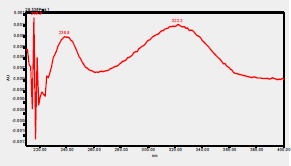
13	Ellagic acid	29.95	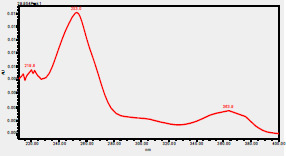
14	Rutin	31.10	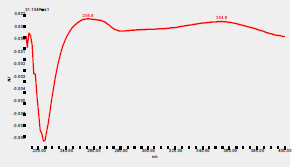
15	Verbascoside	35.15	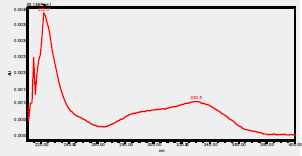
16	Naringin	40.31	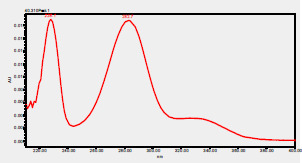
17	Hesparidin	42.41	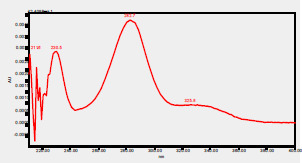
18	Daidzein	48.23	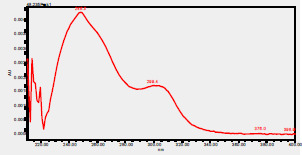
19	Luteolin	50.48	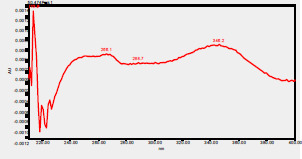
20	Quercetin	50.64	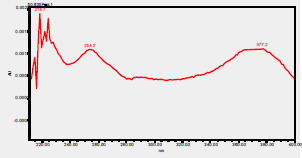
21	Trans-Cinnamic acid	51.53	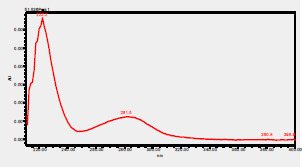
22	Naringenin	54.18	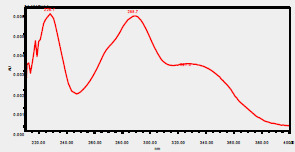
23	Apigenin	54.81	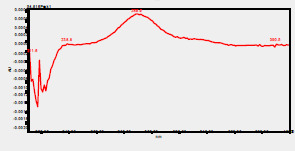
24	Kaempferol	55.0	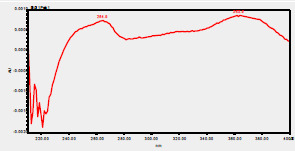
25	Hesperidin	55.62	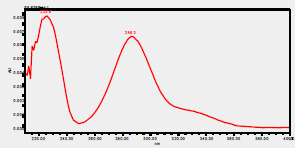
26	Chrysin	63.23	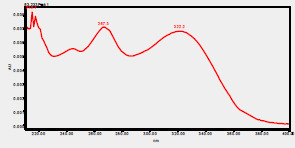
27	Galagnin	64.55	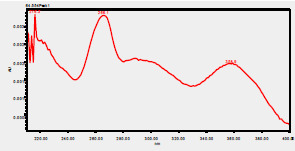

### High-Pressure Liquid Chromatography chromatogram of Ivy leaf extract

**Figure 2:**
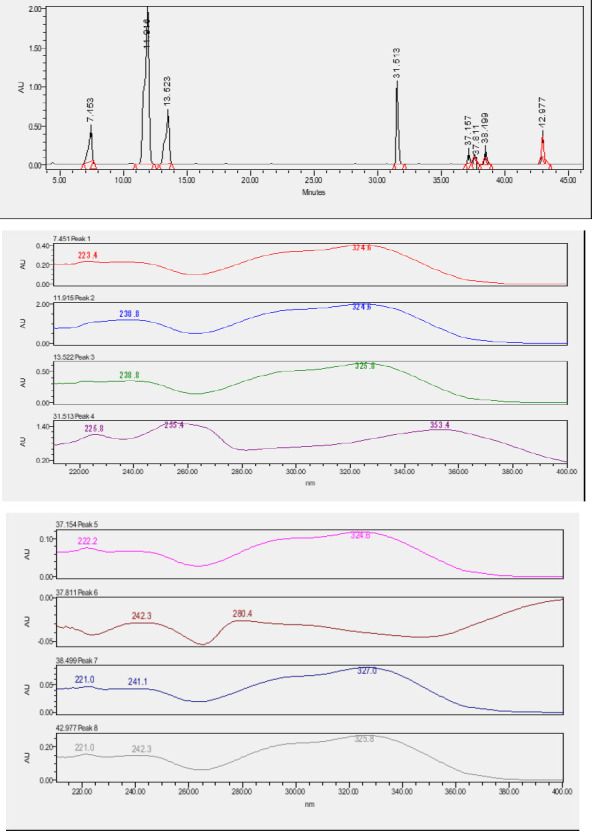
HPLC chromatogram of Ivy leaves extract and UV spectrum of each peak

High-Pressure Liquid Chromatography results were expressed in these selective chromatograms. Figure 2 shows the chromatogram for the Ivy extract. The identification was done through the retention time and wavelengths of the UV spectrum for both standards and samples. Eight peaks were detected, where 3 of them were identified: 4-Hydroxyphenyl acetic acid at 13.52 minutes, rutin at 31.51 minutes, and Hesperidin at 42.97 minutes. 4-Hydroxyphenyl acetic acid is a potent antioxidant^18^, while Rutin has antioxidant and anti-inflammatory effects and it might also offer some protection against cancer and other diseases. Rutin (rutoside, quercetin-3-O-rutinoside) is the glycoside combining the flavonol quercetin and the disaccharide rutinose (α-L-rhamnopyranosylcentrifugation at-(1→6)-β-D-glucopyranose). It is a flavonoid glycoside found in a wide variety of plants^19^. Hesperidin is a flavanone glycoside found in citrus fruits and many other plants, and its aglycone is hesperetin. Hesperidin plays a role in plant defense^20^.

In the literature, many studies investigated the phytochemicals of Ivy leaf extract and found a lot of phytochemicals e.g. triterpene saponins namely α-herein, hedera saponin-C, hederacoside-E, and hederacoside-F, in addition to phenolic acids (caffeic, chlorogenic, neo chlorogenic, caffeoyl-quinic, rosmarinic, dihydroxybenzoic protocatechuic, p-coumaric), and flavonoids (quercetin, kaempferol, rutin, isoquercitrin, astragalin, kaempferol rutinoside)^21^,^2^,^3^,^22^.

## Discussion

To our knowledge, this is the first time to detect the interleukin-6 level using the Real-time reverse-transcriptase method. Previous investigations for the presence of interleukin-6 were carried out through Enzyme Linked Immuno immunosorbent assay (ELISA). Although ELISA is a reliable technique but has certain drawbacks, and the narrow dynamic range is one of them. The dynamic range of an assay refers to the range of concentrations over which the assay can accurately and reliably measure analytes. In the case of ELISAs, this range can be limited, and samples with concentrations outside this range may need to be diluted for accurate measurement. Another challenge associated with ELISA is the matrix interference which may cause false positives or negatives, affecting the assay's sensitivity and specificity^6^,^23^.

Highly specific PCR assays with low cross-reactivity and low detection limits are characteristics of well-designed assays. Real-time PCR has a wide dynamic range and is unaffected by matrix interferences^6^,^24^.

Interleukin-6 is a member of the gp130 cytokine family, which also includes many other cytokines that are useful in the pathophysiology of many diseases. Patients with metabolic disorders have significantly higher levels of TNF-α and IL-6. It is among the proinflammatory cytokines that have higher basal levels than those of normal controls^7^,^25^. Interleukin-6 is one of the important proinflammatory cytokines that play a critical role in the inflammation and immune response. It is secreted in response to tissue injuries, infections, and many inflammatory stimuli. It helps in the synthesis of acute phase reactants, the production of antibodies, as well hematopoiesis^26^,^27^. It is among the cytokines that are responsible for inducing fever during infections. It works on the hypothalamus to increase body temperature as part of the body's defense mechanism to counteract pathogens^1^,^28^. Understanding the effect of IL-6 on inflammation is important for developing therapeutic interventions and gaining insights into the pathophysiology of various immune-related disorders^29^,^30^. An increase in IL-6 gene expression in LPS-pretreated mononuclear phagocytic cells is a promising indicator of our procedure's efficacy in inducing an inflammatory response as shown in Table 4. As shown in Table 3, the extract from Ivy leaves is a strong anti-inflammatory agent that reduces the expression of the IL-6 gene. Its components might prevent specific inflammatory processes, which might reduce the expression of the IL-6 gene. Nevertheless, this is early research, and it's unclear exactly how Ivy leaf extract influences the expression of the IL-6 gene.

The HPLC data provide some insight into the phytochemical composition of Ivy leaf extract (Hedera helix L.). High-Pressure Liquid Chromatography is an effective analytical method for separating and quantification of various compounds in a sample. The identification process involves comparing the retention time and UV spectrum of both standards and samples. This dual approach enhances the accuracy of compound identification. Eight different peaks can be seen on the HPLC chromatogram, which highlights the Ivy extract's complexity. Three compounds—rutin, hesperidin, and 4-hydroxyphenyl acetic acid—have been successfully identified among these peaks. Each of these compounds contributes significantly to the therapeutic and medicinal activities of Ivy leaf extract. 4-Hydroxyphenyl acetic acid is well-known for its antioxidant activity, contributing to the overall free-radical scavenging capacity of the extract. Rutin, a flavonoid, possesses anti-inflammatory and anti-cancer potential. Hesperidin, another flavonoid, is recognized for its cardiovascular benefits.

Accurately identifying the active compounds in Ivy extract is essential to understanding its bioactivities and therapeutic potential because different compounds may have different effects. These results could facilitate more investigation and study into the particular health advantages linked to the compounds found in Ivy extract. Furthermore, this analytical technique offers a consistent way to evaluate the quality of Ivy extract and guarantee that its phytochemical profile is consistent.

## Conclusion

Through the use of Real-Time reverse-transcript PCR, the investigation demonstrated the anti-inflammatory properties of the Hedera helix extract by downregulating IL-6 gene expression. Three identified compounds, one phenolic acid and two flavonoids, are thought to be responsible for these effects. The ethanolic extract contains a large number of polyphenolic compounds and flavonoids. Additional investigation is required to clarify their modes of action.

This study also explored the cytotoxicity and anti-inflammatory effects of Ivy leaf extract, along with its phytochemical composition analyzed via HPLC. Results indicated no significant cytotoxic effects on PMNCs, suggesting that observed reductions in cytokine production were not due to cell death. Moreover, the extract demonstrated anti-inflammatory effects by downregulating IL-6 gene expression, a novel discovery using Real-Time PCR. HPLC analysis identified various beneficial compounds such as rutin and hesperidin, known for their antioxidant and anti-inflammatory properties. The study underscored the limitations of traditional IL-6 detection methods and underscored the advantages of Real-Time PCR. These findings enhance our understanding of Ivy extract's medicinal potential, yet further research is warranted to unravel its mechanisms and clinical applications. Continued exploration of Ivy extract's phytochemical composition may unveil novel bioactive compounds with therapeutic benefits.
